# Association between osteoporosis and benign paroxysmal positional vertigo: a systematic review

**DOI:** 10.1186/1471-2377-14-110

**Published:** 2014-05-20

**Authors:** Shudong Yu, Fenye Liu, Zhixin Cheng, Qirong Wang

**Affiliations:** 1 Department of Otolaryngology, Shandong Qianfoshan Hospital, 16766 Jingshi Road, Jinan 250014, PR China; 2 Department of Traditional Chinese Medicine, Shandong Provincial Hospital affiliated to Shandong University, Jinan, PR China; 3 Department of Vascular Surgery, Shandong Traditional Chinese Medicine Hospital, Jinan, PR China

**Keywords:** Osteoporosis, Osteopenia, Benign paroxysmal positional vertigo, Systematic review

## Abstract

**Background:**

Increasing recent evidence has implicated osteoporosis as a risk factor for benign paroxysmal positional vertigo (BPPV). We conducted a systematic review to examine the association between osteoporosis and BPPV.

**Methods:**

Four electronic databases (PubMed, EMBASE, Cochrane Library, and the China Network Knowledge Infrastructure) were searched to identify all papers, published in either English or Chinese, examining the association between osteoporosis (osteopenia) and BPPV.

**Results:**

Seven studies were eligible for analysis, though these studies included some weaknesses. Most of the studies demonstrated a correlation between osteoporosis (osteopenia) and the occurrence and recurrence of BPPV, especially in older women. Patients with osteoporosis may require more canalith-repositioning procedures.

**Conclusions:**

This systematic review provides insight into currently available evidence and elucidates the possible existence of an association between BPPV and osteoporosis (osteopenia). However, the evidence supporting that conclusion is not strong, and further studies are needed to clarify the association between these conditions.

## Background

Dizziness and vertigo are among the most frequently encountered symptoms in primary care. Benign paroxysmal positional vertigo (BPPV) is the most commonly diagnosed type of vertigo, and is characterized by short-duration vertigo, nausea and/or positional nystagmus associated with changes in head position. One epidemiological study of the general population in Germany conducted by von Brevern and his colleagues found that 2.4% of the population (3.2% of women, 1.6% of men) experienced BPPV at some time during their lives [1]. Posterior canal BPPV is the most common type, accounting for about 90% of cases, while lateral canal BPPV accounts for about 8% [2]. In rare instances, the anterior canal or multiple canals might be involved [3]. BPPV is thought to be caused by the presence of cupulolithiasis or canalithiasis in one or more semicircular canals. However, its exact etiology is unknown and, in most cases, cannot be identified. It may also be secondary to various other conditions, including head trauma [4], labyrinthitis [5], Meniere’s disease [6,7], position during bed rest [8], and migraine [9]. Increasing recent evidence has implicated osteoporosis as a risk factor for BPPV, suggesting that medications used to treat osteoporosis may also help to prevent the occurrence and recurrence of BPPV. It is therefore important to establish the nature of the relationship between osteoporosis and BPPV. The aim of this study was to conduct the first systematic review to examine the association between osteoporosis and BPPV.

## Methods

This study was granted an exception from ethics approval by the Ethics Committee of Shandong Qianfoshan Hospital.

### Data sources

This review included studies published in English or Chinese that examined the association between osteoporosis (osteopenia) and BPPV. The databases searched were PubMed (1966–2013), EMBASE (1974–2013), the Cochrane Library (Issue 3, 2013) and the China Network Knowledge Infrastructure (1979–2013). All searches were completed by November 2013. Search terms included “osteoporosis”, “osteopenia” combined with “benign paroxysmal positional vertigo”, “benign positional vertigo”, “BPPV”, and “BPV”. Two independent reviewers (SY and FL) evaluated the titles and abstracts of all the studies identified in the initial search to locate any potentially relevant studies. The full texts of studies identified as potentially relevant by either reviewer were then evaluated in duplicate.

### Inclusion and exclusion criteria

Studies were eligible if they met the following criteria: studies examined the associations between osteoporosis (osteopenia) and BPPV. In cases of multiple publications of the same or overlapping cohorts, only the studies with the largest sample size were included. The following types of studies were excluded: reviews, commentaries, case reports or letters; studies with insufficient reported data and if the relevant information could not be obtained by contacting the authors.

### Data extraction and quality assessment

Hard copies of all articles included were obtained and read in full. The two authors (SY and FL) extracted data according to pre-defined criteria. Discrepancies were noted and discussed between the authors, and resolved by consensus. Data from the articles were validated and extracted using a pre-defined data-extraction form. The following data were recorded for each study: (1) year of publication; (2) age of subjects; (3) number of subjects; (4) study design; (5) percentages of men and women; (6) outcomes evaluated; and (7) authors’ conclusions. Study quality was evaluated using the Newcastle-Ottawa Scale [10] to assess the risk of bias in individual studies. Each study was evaluated for three broad perspectives using the ‘star system’: (1) selection of study groups; (2) comparability of groups; and (3) ascertainment of the outcome of interest. Study quality was graded as poor (1–3 stars), intermediate (4–6 stars) or high (7–9 stars). Disagreements were resolved by discussions between all authors.

## Results

### Trial selection

**Figure 1:**
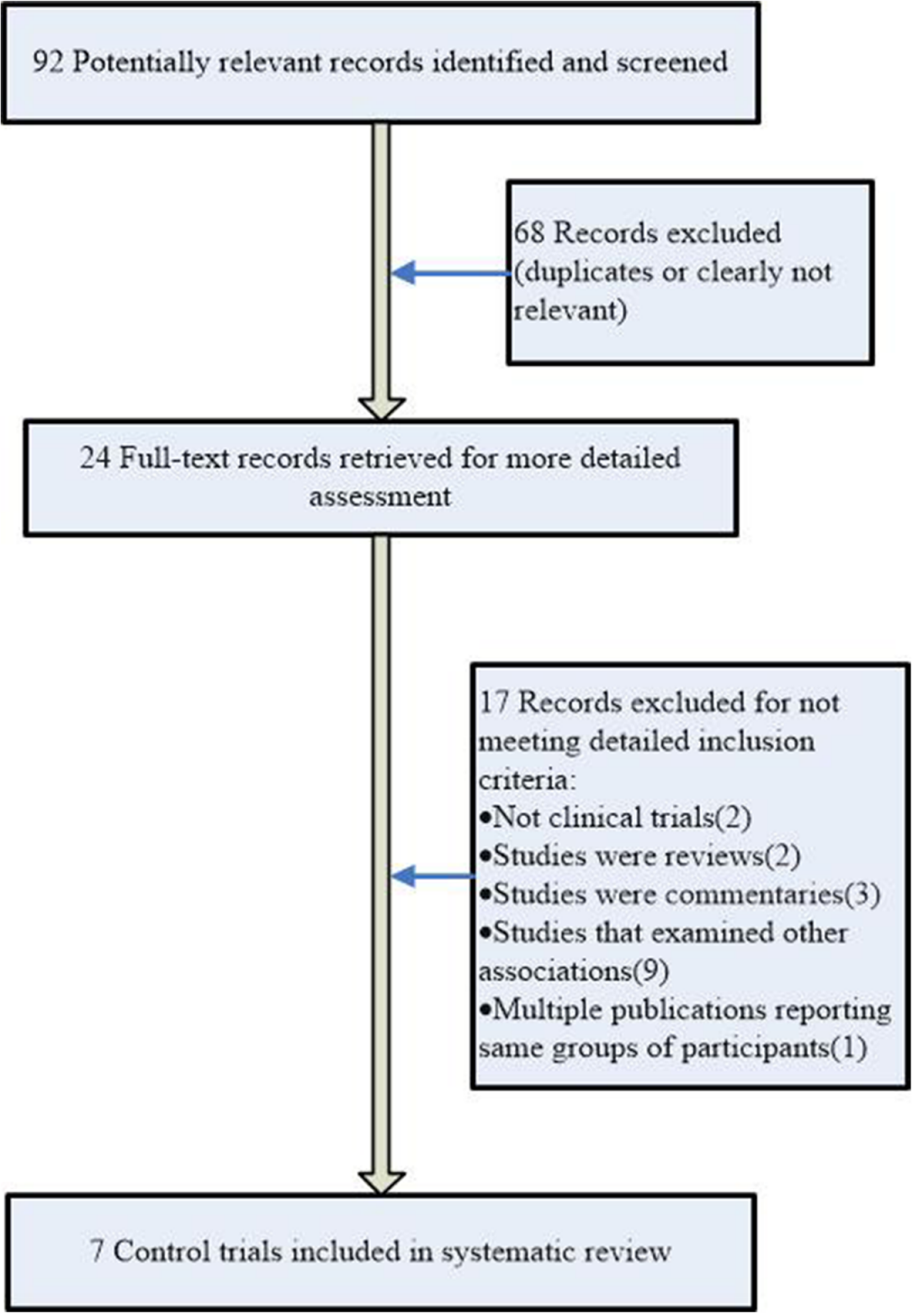
Flow chart depicting the method of study selection.

After screening all titles, abstracts and full texts, the initial search strategy retrieved 92 publications for possible inclusion in the systematic review. Among these 92 publications, seven publications met the inclusion criteria and were retrieved for more in-depth evaluation (Figure 1).

### Description of studies

These seven papers (Table 1) represented seven unique studies, each of which reported an analysis of the association between osteoporosis (osteopenia) and BPPV. Two of the seven studies were conducted in Korea [11,12], two in the United States [13,14], one in Switzerland [15], one in Japan [16], and one in Italy [17]. Two of the seven studies were cohort studies [11,14] and five were case–control studies. One of the seven was a retrospective study [13] and the others were prospective studies. These seven clinical studies included 1,631 BPPV patients and 402 healthy controls. All studies were published in English. None of the trials were randomized controlled trials (RCTs). Because of heterogeneity in study design, data collection, data presentation and lack of RCTs, we did not pool summary data in a meta-analysis.

**Table 1:** Summary of the seven studies that investigated the association between osteoporosis (osteopenia) and BPPV

**Study**	**Age range (Mean ± SD)**	**No. of subjects: control**	**Design**	**Percentage female and male**	**Outcomes evaluated**	**Conclusions of authors**
Vibert 2003 [15]	50 to 85(69 ± 9.2)	32(BPPV):83(Healthy controls)	Case–control study	All were Female	BMD	The apparent correlation between BPPV and osteopenia or osteoporosis
Jang2009 [11]	20 to 69	78(BPPV):117 (Healthy controls)	Cohort studies	All were Female	BMD, The number of canalith repositioning maneuvers, The presence of recurrence	Patients with BPPV had lower BMD values compared with control subjects, and patients with low BMD values showed a significant increase in the number of canalith repositioning maneuvers required and the recurrence rate
Jeong2009 [12]	29 to 90 years (59.8 ± 12.5 in test group, 56.3 ± 8.6 in control group)	209(BPPV):202(Healthy controls)	Case–control study	Female: Male in BPPV Group (142:67), in Control (96:106)	BMD	Decreased BMD score both in women and in men with BBPV, compared with that in controls.
Mikulec2010 [13]	51 to 80	143(BPPV):117(Controls that with symmetric sensorineural hearing loss and without known vestibular problems)	Case–control study	All were Female	The presence or absence of osteoporosis	There was a negative association between BPPV and treated osteoporosis in women aged 51 to 60 years, and a trend towards a negative association for women aged 61–70 years and for the group as a whole. Osteoporosis, or the medication used to treat it, may provide protection against BPPV.
Yamanaka2013 [16]	50 to 88 (63.7 ± 7.40)	39(Recurrence free): 9 (Single recurrence):13(Multiple recurrence)	Case–control study	All were Female	BMD	Osteoporosis is a risk factor for BPPV recurrence. The prognosis of BPPV might be clinically predicted by BMD reduction.
Parham2013 [14]	49 to 81 (66.9 ± 1.8)	16(BPPV):13(osteopenia/osteoporosis)	Cohort studies	All were Female	BMD. Calcium, Vit D, 25(OH)D3, and Serum Markers of Bone Turnover (sNTX, P1NP)	Postmenopausal women with BPPV have a high prevalence of osteopenia/osteoporosis, and postmenopausal women with osteopenia/osteoporosis have higher than expected prevalence of BPPV. Levels of biochemical markers of bone turnover correlate with presence of BPPV but not Ca or Vit D.
Stefano2013 [17]	65 to 95(72.9 ± 6.14)	1092(BPPV):13(BPPV with osteopenia/osteoporosis)	Multicenter Case–control study	Female: Male(685:407)	Risk of recurrence	Combine with two or more comorbidities (hypertension, diabetes, osteoarthrosis), osteoporosis further increases the risk of relapsing BPPV

*SD* standard deviation; *BMD* bone mineral density; *CRP* canalith-repositioning maneuvers, *sNTX* amino-terminal telopeptides of collagen; *P1NP* amino-terminal propeptide of protocollagen type I.

### Study quality

As shown in Tables 2 and 3, two studies [12,17] were graded as ‘high quality’ and five studies [11,13-16] were graded as ‘intermediate quality’. No studies were graded as ‘poor’. The two cohort studies had inadequate follow-up. None of the five case–control studies reported the non-response rate or used the general community as controls. There were particular weaknesses in terms of outcome ascertainment and the representative nature of the cases.

**Table 2:** Methodological quality assessment of cohort studies using the Newcastle-Ottawa Scale

**Study**	**Jang 2009**[11]	**Parham 2013**[14]
SELECTION		
Representativeness of the exposed cohort	☆	☆
Selection of the non-exposed cohort	☆	☆
Ascertainment of exposure	☆	☆
Demonstration that outcome of interest was not present at start of study	☆	☆
COMPARABILITY		
Comparability of cohorts on the basis of the design or analysis	☆	☆
EXPOSURE		
Ascertainment of exposure	☆	☆
Was follow-up long enough for outcomes to occur		
Adequacy of follow up of cohorts		
Total number of star	5	5

Study quality was graded as poor (1–3 stars), intermediate (4–6 stars) or high (7–9 stars).

**Table 3:** Methodological quality assessment of case–control studies using the Newcastle-Ottawa Scale

**Study**	**Vibert 2003**[15]	**Jeong 2009**[12]	**Mikulec 2010**[13]	**Yamanaka 2013**[16]	**Stefano 2013**[17]
SELECTION					
Is the case definition adequate?	☆	☆	☆	☆	☆
Representativeness of the cases		☆	☆		☆
Selection of controls					
Definition of controls	☆	☆	☆	☆	☆
COMPARABILITY					
Comparability of cases and controls on the basis of the design or analysis	☆☆	☆☆	☆	☆☆	☆☆
EXPOSURE					
Ascertainment of exposure	☆	☆		☆	☆
Same method of ascertainment for cases and controls	☆	☆	☆	☆	☆
Non-response rate					
Total number of star	6	7	5	6	7

Study quality was graded as poor (1–3 stars), intermediate (4–6 stars) or high (7–9 stars).

### Study findings

Five clinical studies [11,12,14-16] investigated the association between bone mineral density (BMD) and BPPV. BMD provides a measure of the amount of calcium in areas of the bones, and is used as an indirect clinical indicator of osteoporosis. All of these five studies demonstrated an apparent correlation between BPPV and decreased BMD score; four studies [11,12,14,15] found an association between the occurrence of BPPV and osteoporosis, two [11,16] reported that the prognosis of BPPV might be clinically predicted by BMD reduction, and one [13] reported a negative association between BPPV and treated osteoporosis in women. These results suggest that osteoporosis, or the medication used to treat it, might provide protection against BPPV. In terms of age, one study [15] demonstrated more obvious correlations between BPPV and decreased BMD score in men in their 50s and 70s, but not in their 60s, while another study [11] found obvious correlations in all age groups.

Osteoporosis is common in women after menopause. Among the seven clinical studies analyzed, the study subjects in four [13-16] were postmenopausal women (older women), another study [11] included adult women (about half of them were postmenopausal), while the remaining two studies [12,17] included adult men and women. However, even in these adults, the incidence of osteoporosis was higher in women than in men, with female:male ratios of about 2:1 in these two studies. Adequate intakes of calcium and vitamin D are important foundations for maintaining bone density and strength. Vitamin D deficiency may contribute to the development of osteoporosis. However, one study [14] found no correlation between serum vitamin D levels and the presence of BPPV.

Recurrence of BPPV is a frequent problem. Two studies [11,16] found that the recurrence rate was significantly higher in patients with osteoporosis compared with patients with normal bone mass; furthermore, the frequency of BPPV recurrence increased as BMD decreased. Another study [12] also found that BMD was lower in patients with recurrent BPPV compared with patients with de novo BPPV, but only in women older than 45 years, and not in men or younger women. One study [17] found that osteoporosis was related to an increased risk of relapsing BPPV when it was combined with comorbidities such as hypertension, diabetes, or osteoarthrosis.

The canalith-repositioning procedure (CRP) is currently the main treatment for BPPV. Some patients may require several CRPs, and one study [11] found that patients with lower BMD values underwent significantly more CRPs.

Biochemical markers of bone turnover provide clinically useful evidence of the normal and pathologic processes that reflect bone cell activity in the skeleton. The assessment of bone turnover markers has been proposed to supplement BMD measurement in the diagnosis of osteoporosis. Only one study [14] assessed the association between biochemical markers of bone turnover and BPPV, and found that patients with BPPV had higher amino-terminal propeptide of protocollagen type I levels, and that the level of biochemical markers of bone turnover correlated with the presence of BPPV. However, this study [14] also found no apparent correlation between serum ionized calcium and the presence of BPPV.

## Discussion

This systematic review aimed to select all studies that examined the association between osteoporosis (osteopenia) and BPPV. A total of seven studies were retrieved and analyzed. The results indicate that BPPV may be associated with osteoporosis or osteopenia, and that the medications used to treat these conditions may be able to prevent the occurrence and recurrence of BPPV.

BPPV is the single most common cause of vertigo. CRP provides an effective treatment for most BPPV patients, but the condition has a high recurrence rate; one study found a recurrence rate for BPPV of about 27% in the first 6 months [18]. It is therefore important to gather information to help prevent the occurrence and recurrence of BPPV. This systematic review elucidated the association between osteoporosis and BPPV, and indicated that medications used to treat osteoporosis (such as calcium preparations) may help to prevent the occurrence and recurrence of BPPV.

This review was limited by the fact that the included studies were cohort studies and case–control studies (low evidence), mostly graded as ‘intermediate quality’, and no RCTs (highest evidence) were identified. Studies with a lower level of evidence have a higher risk of bias. The association between osteoporosis and BPPV is better suited to investigation using long-term, multicenter, epidemiological studies. Although one study [17] was a multicenter, observational study, it was not a long-term, epidemiological study, and observational studies are subject to selection, outcome and measurement biases. Further, long-term, multicenter, epidemiological studies or RCTs are therefore needed to confirm the current conclusions.

The subjects in most studies were women, especially older women. The prevalence of BPPV has been shown to be higher in women, and between the ages of 41 and 60 years [19]. Similarly, changes in hormone levels between these ages lead to osteoporosis/osteopenia. One experimental study [20] found ultrastructural modifications of the otoconia in terms of their aspect, size and density in ovariectomized osteopenic/osteoporotic female adult rats. This mechanism may help to explain the higher prevalence of BPPV in women. However, BPPV can also occur at other ages and can occur in men, and the role of osteoporosis in the occurrence of BPPV in these groups remains unanswered. Another experimental study [21] showed that degeneration of the otoconia and linking filaments increased with age in rats, suggesting the existence of an age-related mechanism.

There were several limitations to our study. First, we were unable to perform a meta-analysis because of the lack of RCTs included in this review. Second, only seven relevant studies were identified, with only a few providing evidence for the various outcomes. Third, we limited our search to reports written in Chinese and English, and it is possible that we missed relevant studies published in other languages; however, this would have been beyond the language skills of our team members, though future studies should consider broadening the scope of included papers.

## Conclusions

In conclusion, this systematic review provides an insight into the currently available evidence regarding an association between osteoporosis and BPPV. However, although analysis of the available studies suggests a possible association between BPPV and osteoporosis (osteopenia), the evidence to support that conclusion remains weak, and further studies are needed to confirm the relationship between these conditions.

## Competing interests

The authors declare that they have no competing interests.

## Authors’ contributions

QW designed and prepared this study. SY and FL extracted data and wrote the manuscript. ZC edited and revised the manuscript. All authors read and approved the final manuscript.

## Authors’ information

Shudong Yu and Fenye Liu are co-first authors.

## Pre-publication history

The pre-publication history for this paper can be accessed here:

http://www.biomedcentral.com/1471-2377/14/110/prepub
